# Generated cultural heritage question–answer dataset: Durga in multi-dimensional perspectives

**DOI:** 10.1016/j.dib.2026.112495

**Published:** 2026-01-20

**Authors:** Tri Lathif Mardi Suryanto, Aji Prasetya Wibawa, Hariyono, Andrew Nafalski, Gulsun Kurubacak Çakır

**Affiliations:** a Department of Electrical Engineering and Informatics, Faculty of Engineering, Universitas Negeri Malang, Jl. Semarang no. 5, Malang 65145, Indonesia; b Department of Information System, Faculty of Computer Science, Universitas Pembangunan Nasional Veteran Jawa Timur, Rungku Madya, Surabaya 60294, Indonesia; c Department of History, Faculty of Social Science, Universitas Negeri Malang, Jl. Semarang No. 5, Malang 65145, Indonesia; d UniSA Education Futures, School of Engineering, University of South Australia SCT2-39 Mawson Lakes Campus, Adelaide, South Australia 5095, Australia; e Department of Journalism, College of Communication, Ankara Haci Bayram Veli University, Türkiye

**Keywords:** Generative Question-Answering, NLP, Low-resource domains, Cultural heritage, Digital humanities

## Abstract

This dataset presents a valuable compilation of question–answer (QA) pairs derived from cultural texts and sources related to Durga mythology. A total of 21,395 QA pairs, encompassing textual materials such as scriptures, ritual narratives, temple inscriptions, and traditional storytelling records. Each entry includes the source reference, question, and corresponding answer, provided in a structured format compatible with Excel for seamless integration into downstream natural language processing (NLP) tasks. Data collection involved manual curation and annotation by domain experts, followed by preprocessing steps including text normalization, duplication removal, and verification of factual and contextual accuracy. The dataset is designed to support generative QA models, culturally aware chatbots, and digital preservation of heritage knowledge. It is particularly valuable for research in AI-driven cultural applications, educational tools, and digital humanities initiatives aiming to bridge traditional knowledge with computational methods. Researchers and practitioners may utilize the dataset for training generative models, creating interactive educational platforms, developing culturally sensitive AI agents, and supporting comparative studies in cross-cultural heritage. This openly accessible resource adheres to ethical standards, with proper attribution to source materials, and provides a foundational asset for both academic research and applied development in culturally informed artificial intelligence.

Specifications Table

**Table:** 

Subject	Computer Sciences
Specific subject area	Natural Language Processing, Chatbot, Question-Answering, Cultural Heritage and History Corpus.
Type of data	Text QA pairs, Table.
Data collection	Manual curation, crowdsourcing, web scraping, expert validation.
Data source location	Malang, Indonesia.
Data accessibility	Repository name: Mendeley Data Data identification number: 10.17632/6hcx53kywr.1 Direct URL to data: https://data.mendeley.com/datasets/6hcx53kywr/1 Instructions for accessing these data: Instructions for accessing these data: Click on the direct URL, then use the ‘Download all files’ option in the upper-right corner to obtain the full Excel dataset. No login or special permission is request
Related research article	[1] T. L. M. Suryanto, A. P. Wibawa, H. Hariyono, and A. Nafalski, “Comparative Performance of Transformer Models for Cultural Heritage in NLP Tasks,” Adv. Sustain. Sci. Eng. Technol., vol. 7, no. 1, p. 0250115, Jan. 2025, doi:10.26877/asset.v7i1.1211.

## 1 Value of the Data

• This dataset is unique in presenting domain-specific QA pairs on the topic of Durga as an international and cross-religious issue, offering a comparative perspective across cultures and religious traditions.
• It can be reused to train generative QA models that are applicable not only in the Indonesian context but also within global domains, including Hinduism, Buddhism, and modern academic interpretations.
• The dataset is relevant for researchers in NLP, history, anthropology, and religious studies to explore Durga narratives across diverse traditions.
• It contributes to research on interfaith dialogue and cross-cultural understanding by leveraging AI-driven text-based approaches.
• This corpus represents a valuable dataset for a low-resource cultural heritage domain, addressing the lack of structured question–answer pairs.
• It enables benchmarking and evaluation of domain-adapted QA systems, fostering methodological advances in handling culturally datasets.

## 2 Background

The representation of Durga exhibits considerable diversity across countries, highlighting the need for cross-cultural research to capture its multifaceted meanings. In India, Nepal, Bangladesh, and Sri Lanka, Durga is portrayed through rituals, festivals, and visual arts, each carrying distinct religious and philosophical connotations [2]. In contrast, in Indonesia, Thailand, Cambodia, and Myanmar, Durga is adapted through temples, dance performances, and traditional puppet theatre [3]. Developing such a dataset not only serves documentation purposes but also provides a foundation for comparative studies and cross-cultural knowledge transfer.

Digital preservation presents another critical challenge, as manuscripts, sculptures, paintings, and ritual texts related to Durga are often vulnerable to deterioration, natural disasters, or limited conservation resources. Differences in conservation policies and capacities across countries result in significant gaps in data accessibility [4]. This dataset was developed to address the increasing demand for structured cultural heritage resources that document the diverse representations of Durga in Asia, as well as global communities cultural heritage and history. The dataset was constructed following principles from digital humanities [5], natural language processing (NLP) [6], and hybrid deep transformer architectures combined with hyperparameter optimization [7], with annotated data enabling tasks such as visual recognition, semantic analysis, cross-cultural comparison, and AI-driven educational applications.

## 3 Data Description

The narratives of Goddess Durga, Durga remains a living narrative across South and Southeast Asia including India, Nepal, Bangladesh, Sri Lanka, Indonesia, Thailand, Cambodia, and Myanmar as well as within global Hindu diasporas, reflecting her role as an enduring international and cross-cultural figure. The data set presented an Excel file that is publicly accessible in the Mendeley Data repository: https://data.mendeley.com/datasets/6hcx53kywr/1. The dataset comprises 4,279 rows, each corresponding to a unique entry of cultural heritage knowledge about Durga, accompanied by multiple variations of questions and their associated answers to facilitate natural language processing (NLP) applications.

Table 1 presents the dataset structure, which is organized at a single folder level with one primary file, Question-Answer Pairs Goddess Durga. This Excel file contains unique entries across seven columns (ID, Answer, Question 1–5), where each answer is paired with five question variations, resulting in 21,395 questions. The compact format, combining factual answers, diverse question formulations, and thematic metadata, ensures ease of use and direct integration into NLP workflows for cultural heritage and question-answering research.

**Table 1:** Dataset example.

ID	Answer	Question 1	Question 2	Question 3	Question 4	Question 5
1	Durga in India was originally depicted as a beautiful, multi-armed Goddess of War.	How Durga in India was originally depicted in Hindu tradition	Who is the Goddess who was originally known as the Goddess of War in India and is depicted as having many arms?	In Indian mythology, how was the figure of Goddess Durga initially depicted?	In Indian culture, what was the early representation of Goddess Durga?	What are the physical characteristics of Goddess Durga in India in her early depictions?
…						
4279	In Bali, Pura Dalem Puri and all the Pura Dalem in Pekraman Village are the places where Goddess Durga resides.	Where does Goddess Durga reside in Bali?	What are the places in Bali where Goddess Durga resides?	Why is Pura Dalem Puri important for Goddess Durga?	What is the role of Pura Dalem in Pekraman Village in the worship of Dewi Durga?	What is the relationship between Goddess Durga and Pura Dalem?

The content centers on the mythology, depictions, and worship of Goddess Durga, with a particular emphasis on the cultural context. The Answer column provides factual or descriptive statements, while the Question columns contain variations formulated with diverse interrogatives (e.g., “Who,” “What,” “How,” “Where”). With its structured format and domain-specific focus, this dataset constitutes a high-quality resource for research in question-answering and the development of conversational AI grounded in cultural heritage.

**Fig. 1:**
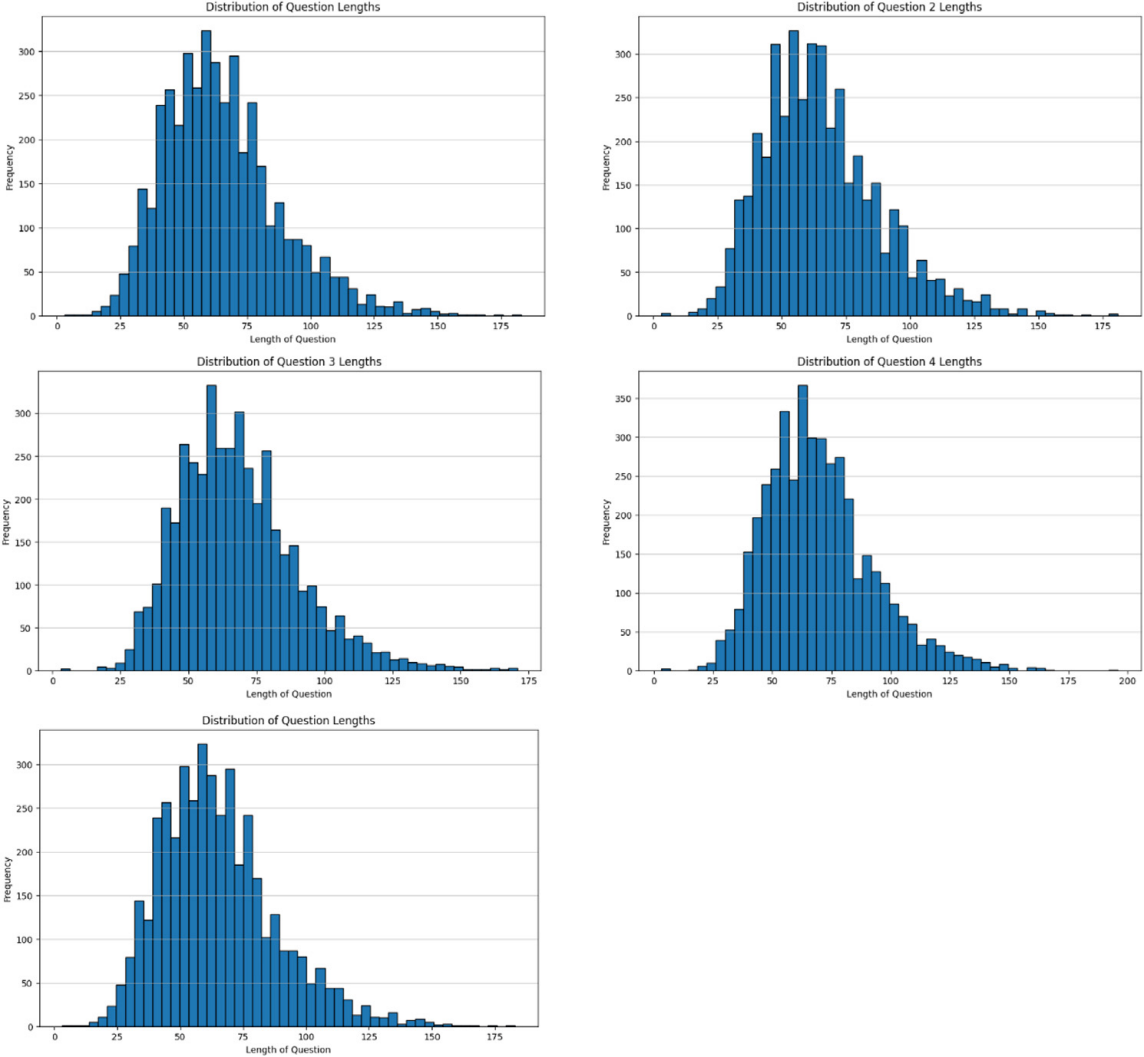
Distribution of question-answering length.

The histogram in Fig. 1, show of question lengths indicates that the majority fall within the 50–70-word range, reflecting their narrative and contextual nature, which is well-suited for evaluating question-answering models on complex texts. The statistical analysis of question lengths demonstrates that the dataset contains 4,279 entries with an average length of approximately 65 tokens, a median of 62, and a range from 3 to 183. This distribution, with 50% of the questions concentrated between 48 and 77 tokens, indicates a balanced structure that avoids domination by excessively short or excessively long entries*.*

**Fig. 2:**
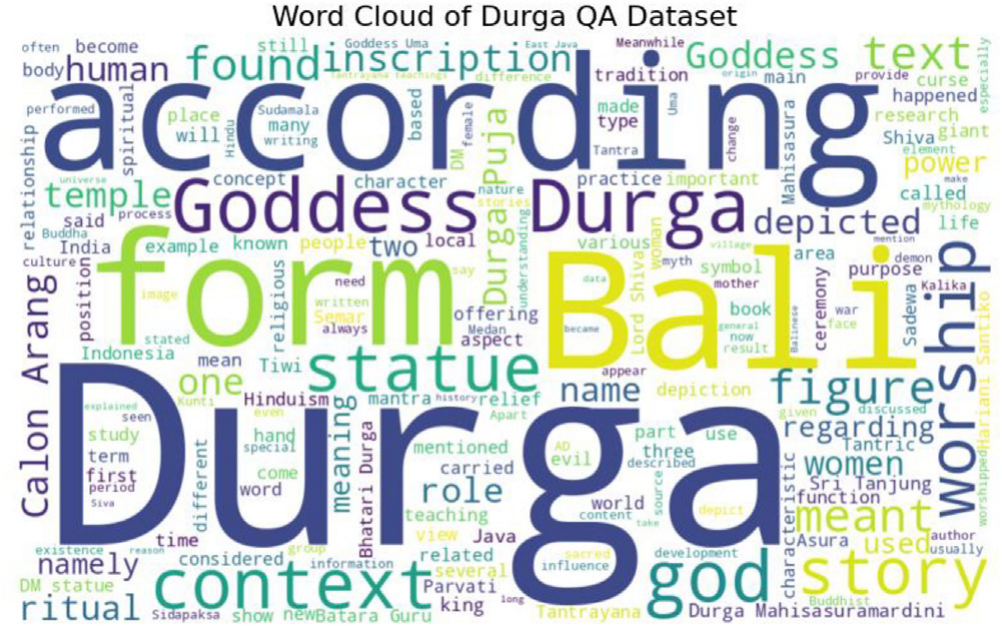
Word cloud of Durga.

In Fig. 2, the word cloud of the Cultural Heritage dataset highlights the dominance of terms such as Durga, Bali, form, statue, goddess, worship, and context, reflecting the centrality of ritual practices, iconography, and religious interpretation in both local and transnational perspectives. The prominence of Bali and Calon Arang underscores the integration of Durga into Balinese cultural narratives, while words like inscription, temple, and ritual signal strong connections to archaeological and textual sources. The coexistence of spiritual, mythological, and scholarly terms (power, meaning, research, teaching) indicates that the dataset spans not only religious devotion but also academic and intercultural interpretations. This lexical distribution confirms that Durga is positioned as a cross-cultural and cross-religious figure, enabling the dataset to serve as a robust foundation for both NLP applications and international cultural heritage research.

## 4 Experimental Design, Materials and Methods

**Fig. 3:**
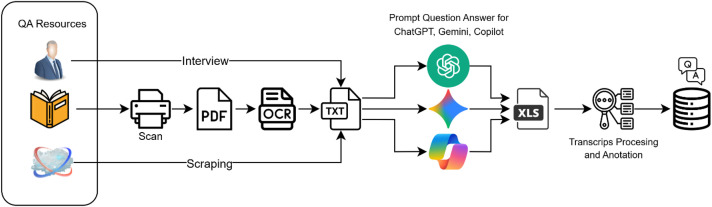
The pipeline from Durga Book to the final structured dataset.

The data collection process was conducted by extracting fundamental knowledge from the Indonesian manuscript and experts from diverse disciplinary backgrounds. Each chapter was digitized through scanning into PDF format and subsequently converted into machine-readable text (.txt) using Optical Character Recognition (OCR) and translated into English. To reduce transcription errors, a multi-stage validation strategy was applied, including manual inspection by domain-aware annotators, random spot-checking to estimate character and word level accuracy, and dual annotator consensus based correction to minimize residual OCR noise. From these English textual sources, question–answer (QA) pairs were systematically constructed, where each factual answer was associated with five distinct question formulations. This design choice reflects the assumption that in real-world communication, a single answer may be elicited by multiple questions. The generation of question variations was supported by large language models, including ChatGPT, Copilot, and Gemini, thereby ensuring linguistic diversity and enhancing the dataset’s suitability for Natural Language Processing (NLP) applications. Question variations were generated using the exact same prompt was used. prompt template that guided large language models to produce semantically equivalent questions with diverse linguistic formulations, show in Fig. 3.

The text preprocessing pipeline involves several sequential steps: converting all text to lowercase, intent, removing punctuation, tokenizing the text into individual words, and filtering out common English stopwords. This process ensures that the resulting text is normalized, noise-free, and ready for subsequent natural language processing tasks. The annotation process was carried out by domain experts. Professor Tom Hoogervorst from Universiteit Leiden and Dr. Grace Leksana from Utrecht University validate the scholarly rigor and cultural authenticity of the dataset, The dataset does not include individual participant responses, except for two expert validations conducted by scholars from Leiden University and Utrecht University to minimize cultural bias. No questionnaire was administered, as the dataset was constructed entirely from textual question–answer pairs, each stored as text data without coded variables or codebook (Table 2).

**Table 2:** Dataset structure.

Column Name	Description	Example
ID	Unique identifier for each entry in the dataset.	01
Answer	Factual text providing information about Goddess Durga, drawn from curated cultural and historical sources.	Each of her arms holds a weapon bestowed by Lord Shiva and other gods during her creation as the Goddess of War and Protector of the gods to defeat her powerful enemy Mahisasura, the buffalo-headed king of the demons
Question 1	First formulation of a question corresponding to the given answer.	What does each arm of Goddess Durga hold in Indian mythology?
Question 2	Second variation of the question is linked to the same answer.	Who gave weapons to Goddess Durga during her creation, and what is the purpose of these weapons?
Question 3	Third variation of the question, designed to capture linguistic diversity.	Who defeated Mahisasura, the buffalo-headed demon king, in Indian mythology, and how?
Question 4	Fourth variation of the question, representing alternative phrasing.	How did Goddess Durga obtain the weapons she wields, and for what purpose?
Question 5	Fifth variation of the question, ensuring multiple ways of querying the same information.	In Indian mythology, what was Goddess Durga created for, and who was the main enemy she had to defeat?

All scripts were developed in Python 3.9 and executed on Windows 11, using libraries such as pandas, numpy, nltk, spacy, regex, matplotlib, seaborn, wordcloud, scikit-learn, and Hugging Face Transformers for data handling, preprocessing, visualization, and model fine-tuning. The experiments were conducted on a system with an Intel(R) Core (TM) i5-8350U CPU @ 1.70 GHz, 8 GB RAM, and a 64-bit x64-based architecture, which provided sufficient resources for efficient data processing and model training.

## Limitations

Several limitations were identified in the construction of this dataset, particularly from a technical and procedural perspective. First, the dataset is limited to the theme of Goddess Durga, restricting its coverage to a specific cultural and religious domain and thereby limiting its generalizability across broader cultural heritage contexts. Second, the dataset is entirely text-based, as answers were extracted from OCR-processed documents and stored in tabular form, without integrating multimodal resources such as iconographic depictions, inscriptions, or ritual recordings that could enrich contextual understanding, dataset focuses on text-based QA construction to establish a validated foundational corpus, while multimodal integration is reserved for future work. Third, the question–answer pairs were generated in a static manner through a combination of manual curation and large language models (ChatGPT, Copilot, Gemini), resulting in fixed textual representations that may not capture the variability and dynamism of real-world questioning strategies. Fourth, although OCR-based digitization was applied and validated through multi-stage verification procedures, residual transcription noise or minor formatting inconsistencies may still persist and affect the precision of downstream processing. Lastly, the absence of metadata enrichment—such as temporal markers, geographic tagging, or speaker annotations—limits the dataset’s capacity for advanced cross-disciplinary analyses. Despite these constraints, the dataset remains a technically sound, well-structured, and replicable resource for advancing research in cultural heritage and Natural Language Processing, particularly in developing and evaluating question-answering systems*.*

## Ethics Statement

This work involved data collection exclusively from publicly available cultural texts and literature, ensuring that no personal or sensitive information was included in the dataset. All materials are derived from published sources, and the dataset does not contain any identifiable personal data. The resource has been fully anonymized and is intended strictly for academic and non-commercial research purposes. The authors confirm that they have read and adhered to the ethical requirements for publication in Data in Brief and that this work does not involve human subjects, animal experiments, or data requiring informed consent.

## CRediT author statement

**Tri Lathif Mardi Suryanto:** Conceptualization, Data Curation, Methodology, Writing. **Aji Prasetya Wibawa:** Supervision, Validation, Project Administration, Writing. **Hariyono:** Resources, Quality Control, Data Verification. **Andrew Nafalski:** Investigation, Technical Support, Code Optimization. **Gulsun Kurubacak Çakır:** Quality Control, Data Verification.

## Data Availability

Mendeley DataQuestion-Answer Pairs Goddess Durga (Original data)
